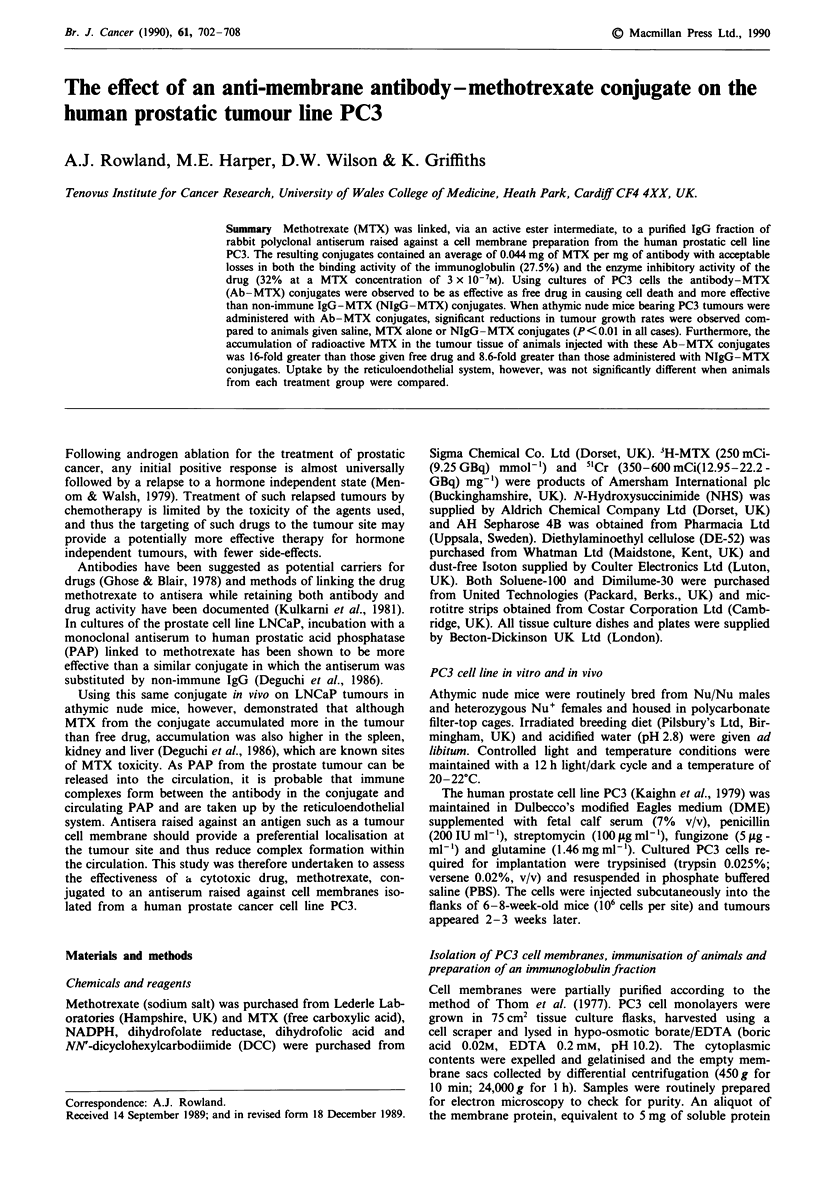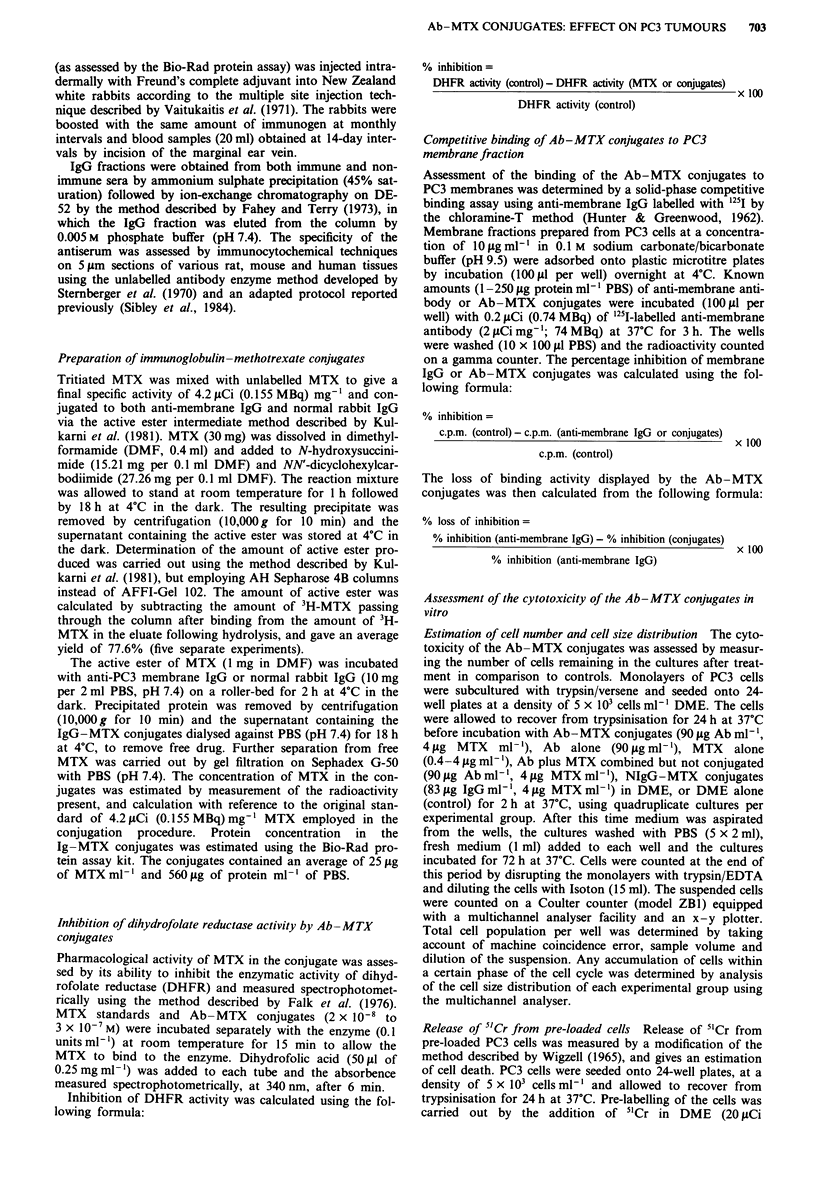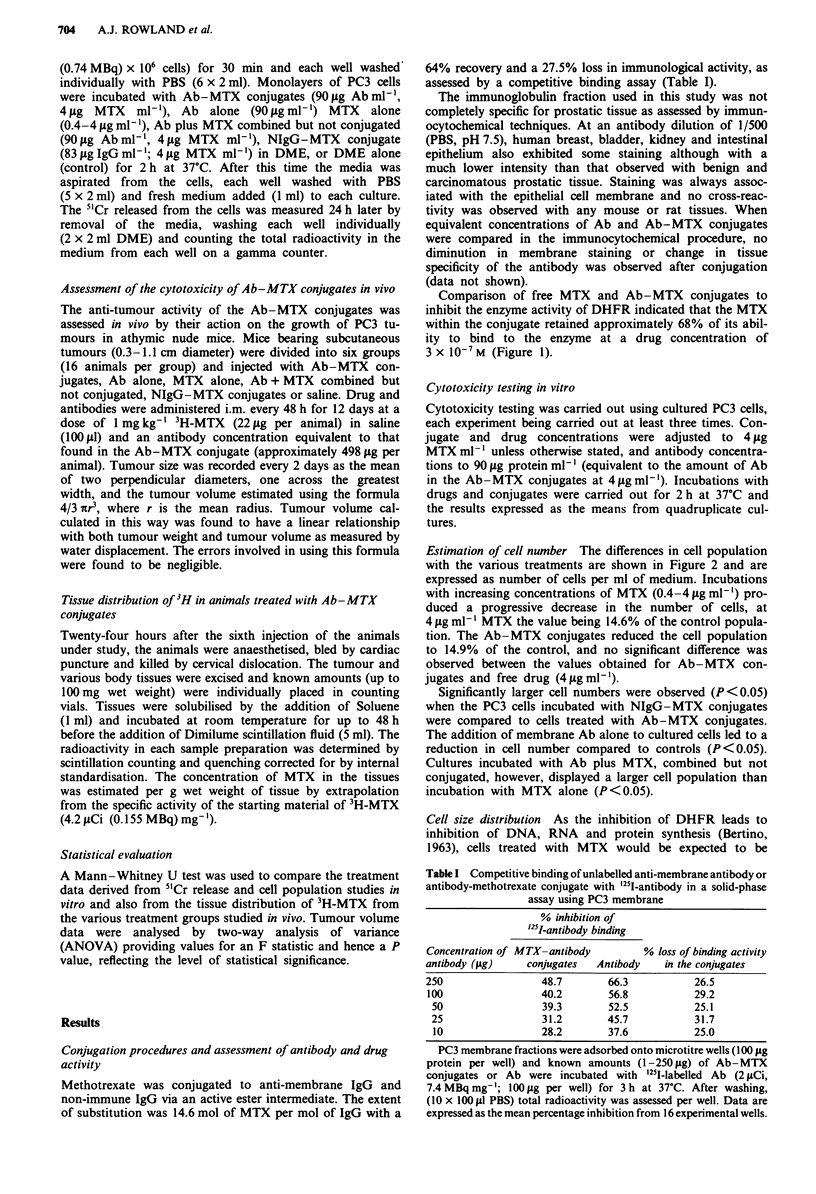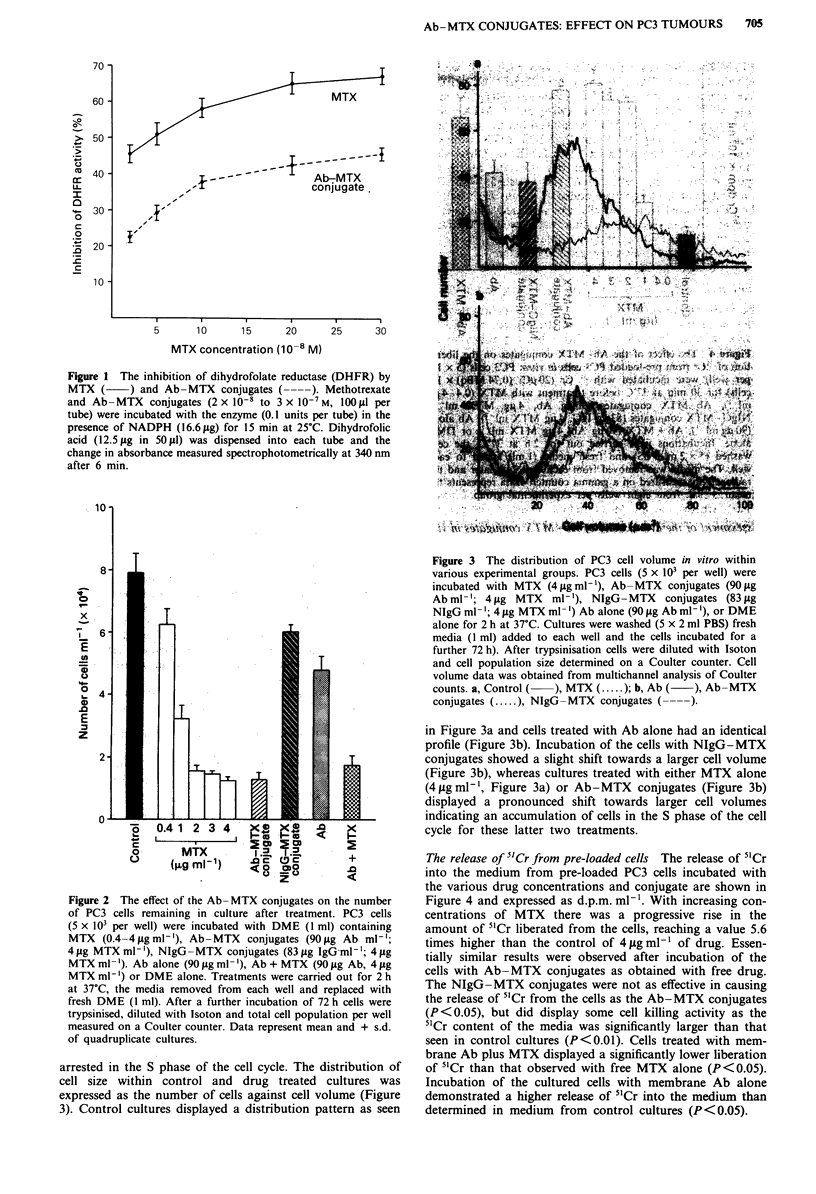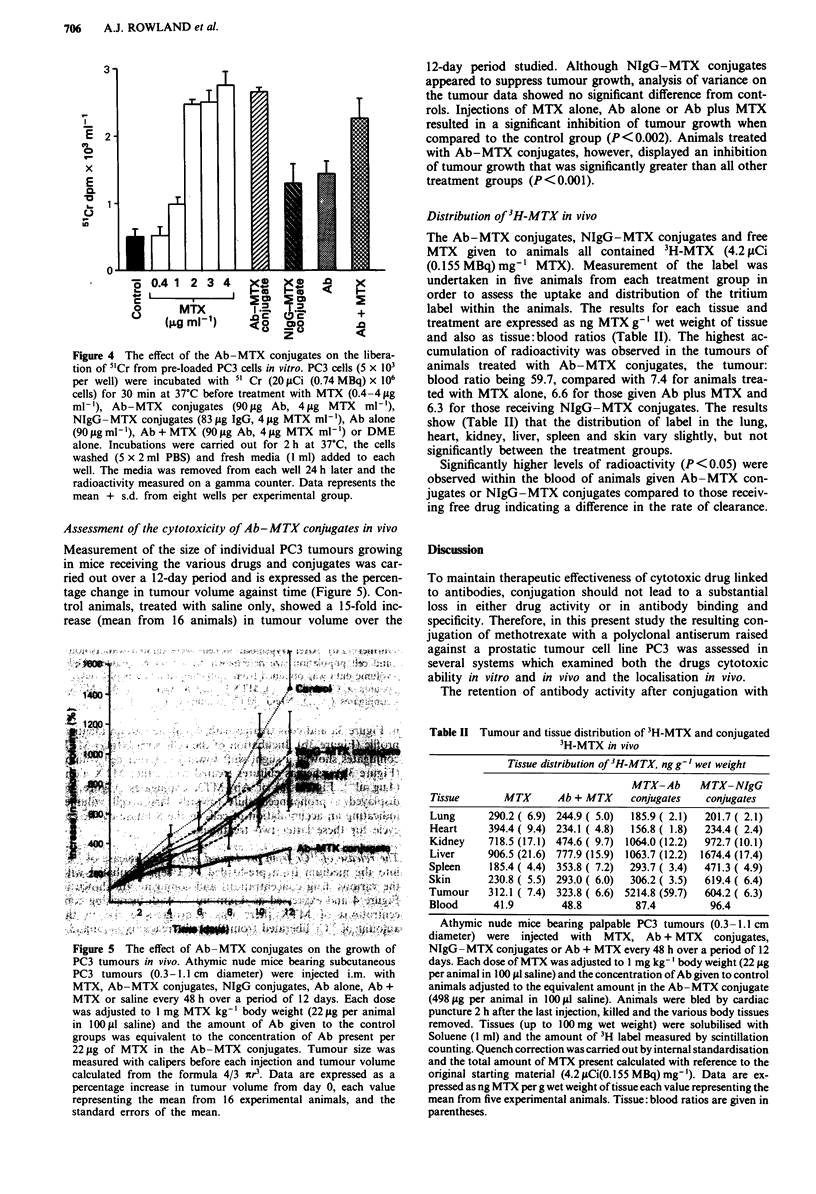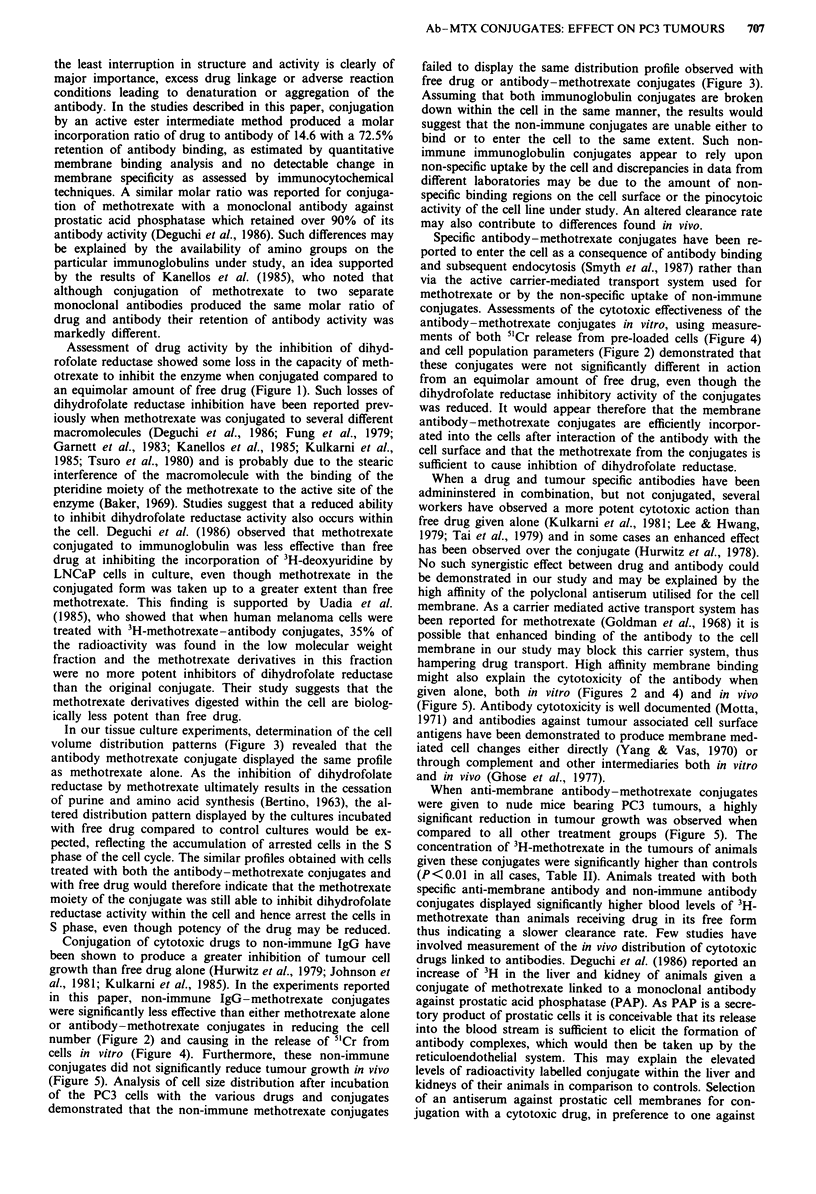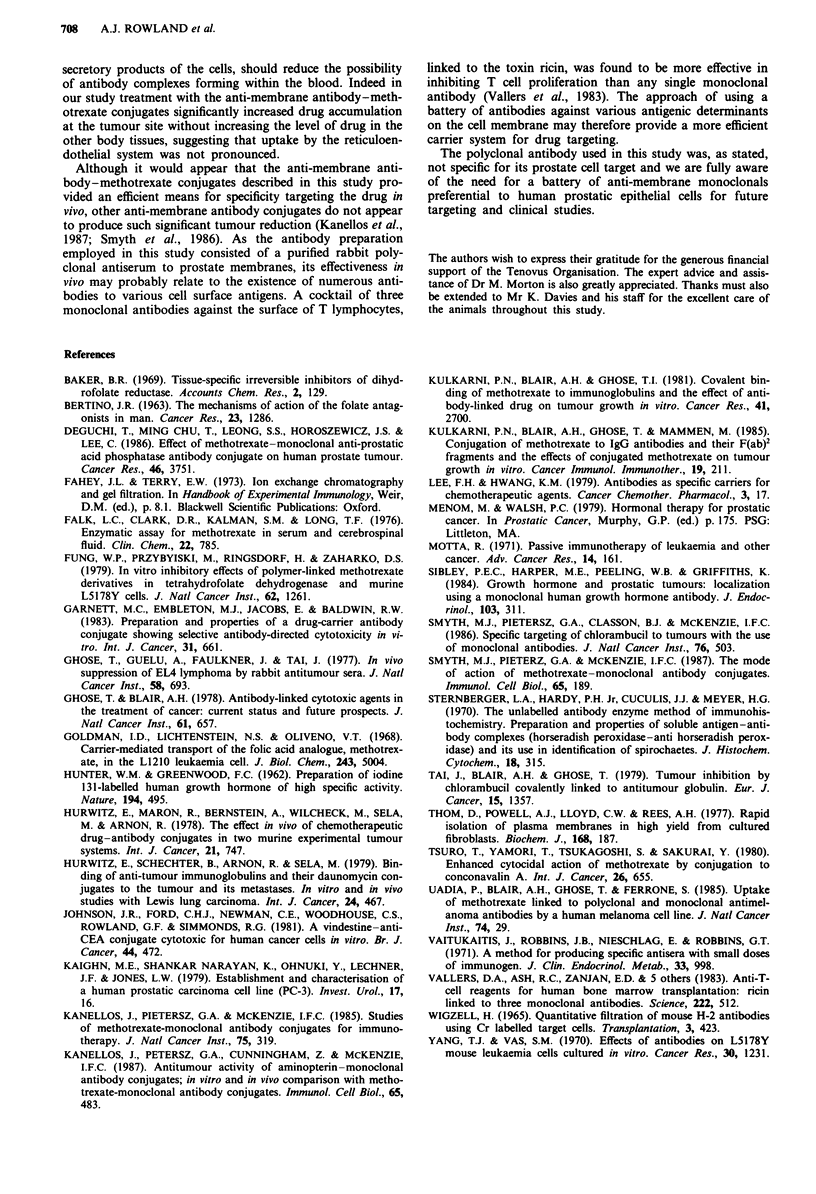# The effect of an anti-membrane antibody-methotrexate conjugate on the human prostatic tumour line PC3.

**DOI:** 10.1038/bjc.1990.158

**Published:** 1990-05

**Authors:** A. J. Rowland, M. E. Harper, D. W. Wilson, K. Griffiths

**Affiliations:** Tenovus Institute for Cancer Research, University of Wales College of Medicine, Heath Park, Cardiff, UK.

## Abstract

Methotrexate (MTX) was linked, via an active ester intermediate, to a purified IgG fraction of rabbit polyclonal antiserum raised against a cell membrane preparation from the human prostatic cell line PC3. The resulting conjugates contained an average of 0.044 mg of MTX per mg of antibody with acceptable losses in both the binding activity of the immunoglobulin (27.5%) and the enzyme inhibitory activity of the drug (32% at a MTX concentration of 3 x 10(-7) M). Using cultures of PC3 cells the antibody-MTX (Ab-MTX) conjugates were observed to be as effective as free drug in causing cell death and more effective than non-immune IgG-MTX (NIgG-MTX) conjugates. When athymic nude mice bearing PC3 tumours were administered with Ab-MTX conjugates, significant reductions in tumour growth rates were observed compared to animals given saline, MTX alone or NIgG-MTX conjugates (P less than 0.01 in all cases). Furthermore, the accumulation of radioactive MTX in the tumour tissue of animals injected with these Ab-MTX conjugates was 16-fold greater than those given free drug and 8.6-fold greater than those administered with NIgG-MTX conjugates. Uptake by the reticuloendothelial system, however, was not significantly different when animals from each treatment group were compared.


Br. J. Cancer (1990), 61, 702-708 © Macmillan Press Ltd., 1990

### The effect of an anti-membrane antibody-methotrexate conjugate on the human prostatic tumour line PC3

A.J. Rowland, M.E. Harper, D.W. Wilson & K. Griffiths

*Tenovus Institute for Cancer Research, University of Wales College of Medicine, Heath Park, Cardiff CF4 4XX, UK.*

**Summary** Methotrexate (MTX) was linked, via an active ester intermediate, to a purified IgG fraction of rabbit polyclonal antiserum raised against a cell membrane preparation from the human prostatic cell line PC3. The resulting conjugates contained an average of 0.044 mg of MTX per mg of antibody with acceptable losses in both the binding activity of the immunoglobulin (27.5%) and the enzyme inhibitory activity of the drug (32% at a MTX concentration of $3 \times 10^{-7}$M). Using cultures of PC3 cells the antibody-MTX (Ab-MTX) conjugates were observed to be as effective as free drug in causing cell death and more effective than non-immune IgG-MTX (NIgG-MTX) conjugates. When athymic nude mice bearing PC3 tumours were administered with Ab-MTX conjugates, significant reductions in tumour growth rates were observed compared to animals given saline, MTX alone or NIgG-MTX conjugates ($P < 0.01$ in all cases). Furthermore, the accumulation of radioactive MTX in the tumour tissue of animals injected with these Ab-MTX conjugates was 16-fold greater than those given free drug and 8.6-fold greater than those administered with NIgG-MTX conjugates. Uptake by the reticuloendothelial system, however, was not significantly different when animals from each treatment group were compared.

Following androgen ablation for the treatment of prostatic cancer, any initial positive response is almost universally followed by a relapse to a hormone independent state (Menom & Walsh, 1979). Treatment of such relapsed tumours by chemotherapy is limited by the toxicity of the agents used, and thus the targeting of such drugs to the tumour site may provide a potentially more effective therapy for hormone independent tumours, with fewer side-effects.

Antibodies have been suggested as potential carriers for drugs (Ghose & Blair, 1978) and methods of linking the drug methotrexate to antisera while retaining both antibody and drug activity have been documented (Kulkarni *et al.*, 1981). In cultures of the prostate cell line LNCaP, incubation with a monoclonal antiserum to human prostatic acid phosphatase (PAP) linked to methotrexate has been shown to be more effective than a similar conjugate in which the antiserum was substituted by non-immune IgG (Deguchi *et al.*, 1986).

Using this same conjugate *in vivo* on LNCaP tumours in athymic nude mice, however, demonstrated that although MTX from the conjugate accumulated more in the tumour than free drug, accumulation was also higher in the spleen, kidney and liver (Deguchi *et al.*, 1986), which are known sites of MTX toxicity. As PAP from the prostate tumour can be released into the circulation, it is probable that immune complexes form between the antibody in the conjugate and circulating PAP and are taken up by the reticuloendothelial system. Antisera raised against an antigen such as a tumour cell membrane should provide a preferential localisation at the tumour site and thus reduce complex formation within the circulation. This study was therefore undertaken to assess the effectiveness of a cytotoxic drug, methotrexate, conjugated to an antiserum raised against cell membranes isolated from a human prostate cancer cell line PC3.

### Materials and methods

### *Chemicals and reagents*

Methotrexate (sodium salt) was purchased from Lederle Laboratories (Hampshire, UK) and MTX (free carboxylic acid), NADPH, dihydrofolate reductase, dihydrofolic acid and *NN'*-dicyclohexylcarbodiimide (DCC) were purchased from Sigma Chemical Co. Ltd (Dorset, UK). $^3$H-MTX (250 mCi-(9.25 GBq) mmol$^{-1}$) and $^{51}$Cr (350-600 mCi(12.95-22.2-GBq) mg$^{-1}$) were products of Amersham International plc (Buckinghamshire, UK). *N*-Hydroxysuccinimide (NHS) was supplied by Aldrich Chemical Company Ltd (Dorset, UK) and AH Sepharose 4B was obtained from Pharmacia Ltd (Uppsala, Sweden). Diethylaminoethyl cellulose (DE-52) was purchased from Whatman Ltd (Maidstone, Kent, UK) and dust-free Isoton supplied by Coulter Electronics Ltd (Luton, UK). Both Soluene-100 and Dimilume-30 were purchased from United Technologies (Packard, Berks., UK) and microtitre strips obtained from Costar Corporation Ltd (Cambridge, UK). All tissue culture dishes and plates were supplied by Becton-Dickinson UK Ltd (London).

### *PC3 cell line in vitro and in vivo*

Athymic nude mice were routinely bred from Nu/Nu males and heterozygous Nu$^+$ females and housed in polycarbonate filter-top cages. Irradiated breeding diet (Pilsbury's Ltd, Birmingham, UK) and acidified water (pH 2.8) were given *ad libitum*. Controlled light and temperature conditions were maintained with a 12 h light/dark cycle and a temperature of 20-22°C.

The human prostate cell line PC3 (Kaighn *et al.*, 1979) was maintained in Dulbecco's modified Eagles medium (DME) supplemented with fetal calf serum (7% v/v), penicillin (200 IU ml$^{-1}$), streptomycin (100 μg ml$^{-1}$), fungizone (5 μg-ml$^{-1}$) and glutamine (1.46 mg ml$^{-1}$). Cultured PC3 cells required for implantation were trypsinised (trypsin 0.025%; versene 0.02%, v/v) and resuspended in phosphate buffered saline (PBS). The cells were injected subcutaneously into the flanks of 6-8-week-old mice ($10^6$ cells per site) and tumours appeared 2-3 weeks later.

### *Isolation of PC3 cell membranes, immunisation of animals and preparation of an immunoglobulin fraction*

Cell membranes were partially purified according to the method of Thom *et al.* (1977). PC3 cell monolayers were grown in 75 cm$^2$ tissue culture flasks, harvested using a cell scraper and lysed in hypo-osmotic borate/EDTA (boric acid 0.02M, EDTA 0.2 mM, pH 10.2). The cytoplasmic contents were expelled and gelatinised and the empty membrane sacs collected by differential centrifugation (450 *g* for 10 min; 24,000 *g* for 1 h). Samples were routinely prepared for electron microscopy to check for purity. An aliquot of the membrane protein, equivalent to 5 mg of soluble protein

Correspondence: A.J. Rowland.
Received 14 September 1989; and in revised form 18 December 1989.

(as assessed by the Bio-Rad protein assay) was injected intradermally with Freund's complete adjuvant into New Zealand white rabbits according to the multiple site injection technique described by Vaitukaitis *et al.* (1971). The rabbits were boosted with the same amount of immunogen at monthly intervals and blood samples (20 ml) obtained at 14-day intervals by incision of the marginal ear vein.

IgG fractions were obtained from both immune and non-immune sera by ammonium sulphate precipitation (45% saturation) followed by ion-exchange chromatography on DE-52 by the method described by Fahey and Terry (1973), in which the IgG fraction was eluted from the column by 0.005 M phosphate buffer (pH 7.4). The specificity of the antiserum was assessed by immunocytochemical techniques on 5 μm sections of various rat, mouse and human tissues using the unlabelled antibody enzyme method developed by Sternberger *et al.* (1970) and an adapted protocol reported previously (Sibley *et al.*, 1984).

*Preparation of immunoglobulin-methotrexate conjugates*

Tritiated MTX was mixed with unlabelled MTX to give a final specific activity of 4.2 μCi (0.155 MBq) $mg^{-1}$ and conjugated to both anti-membrane IgG and normal rabbit IgG via the active ester intermediate method described by Kulkarni *et al.* (1981). MTX (30 mg) was dissolved in dimethylformamide (DMF, 0.4 ml) and added to *N*-hydroxysuccinimide (15.21 mg per 0.1 ml DMF) and *NN'*-dicyclohexylcarbodiimide (27.26 mg per 0.1 ml DMF). The reaction mixture was allowed to stand at room temperature for 1 h followed by 18 h at 4°C in the dark. The resulting precipitate was removed by centrifugation (10,000 *g* for 10 min) and the supernatant containing the active ester was stored at 4°C in the dark. Determination of the amount of active ester produced was carried out using the method described by Kulkarni *et al.* (1981), but employing AH Sepharose 4B columns instead of AFFI-Gel 102. The amount of active ester was calculated by subtracting the amount of $^{3}$H-MTX passing through the column after binding from the amount of $^{3}$H-MTX in the eluate following hydrolysis, and gave an average yield of 77.6% (five separate experiments).

The active ester of MTX (1 mg in DMF) was incubated with anti-PC3 membrane IgG or normal rabbit IgG (10 mg per 2 ml PBS, pH 7.4) on a roller-bed for 2 h at 4°C in the dark. Precipitated protein was removed by centrifugation (10,000 *g* for 10 min) and the supernatant containing the IgG-MTX conjugates dialysed against PBS (pH 7.4) for 18 h at 4°C, to remove free drug. Further separation from free MTX was carried out by gel filtration on Sephadex G-50 with PBS (pH 7.4). The concentration of MTX in the conjugates was estimated by measurement of the radioactivity present, and calculation with reference to the original standard of 4.2 μCi (0.155 MBq) $mg^{-1}$ MTX employed in the conjugation procedure. Protein concentration in the Ig-MTX conjugates was estimated using the Bio-Rad protein assay kit. The conjugates contained an average of 25 μg of MTX $ml^{-1}$ and 560 μg of protein $ml^{-1}$ of PBS.

*Inhibition of dihydrofolate reductase activity by Ab-MTX conjugates*

Pharmacological activity of MTX in the conjugate was assessed by its ability to inhibit the enzymatic activity of dihydrofolate reductase (DHFR) and measured spectrophotometrically using the method described by Falk *et al.* (1976). MTX standards and Ab-MTX conjugates ($2 \times 10^{-8}$ to $3 \times 10^{-7}$ M) were incubated separately with the enzyme (0.1 units $ml^{-1}$) at room temperature for 15 min to allow the MTX to bind to the enzyme. Dihydrofolic acid (50 μl of 0.25 mg $ml^{-1}$) was added to each tube and the absorbence measured spectrophotometrically, at 340 nm, after 6 min.

Inhibition of DHFR activity was calculated using the following formula:

$$\%\ \text{inhibition} = \frac{\text{DHFR activity (control)} - \text{DHFR activity (MTX or conjugates)}}{\text{DHFR activity (control)}} \times 100$$

*Competitive binding of Ab-MTX conjugates to PC3 membrane fraction*

Assessment of the binding of the Ab-MTX conjugates to PC3 membranes was determined by a solid-phase competitive binding assay using anti-membrane IgG labelled with $^{125}$I by the chloramine-T method (Hunter & Greenwood, 1962). Membrane fractions prepared from PC3 cells at a concentration of 10 μg $ml^{-1}$ in 0.1 M sodium carbonate/bicarbonate buffer (pH 9.5) were adsorbed onto plastic microtitre plates by incubation (100 μl per well) overnight at 4°C. Known amounts (1-250 μg protein $ml^{-1}$ PBS) of anti-membrane antibody or Ab-MTX conjugates were incubated (100 μl per well) with 0.2 μCi (0.74 MBq) of $^{125}$I-labelled anti-membrane antibody (2 μCi $mg^{-1}$; 74 MBq) at 37°C for 3 h. The wells were washed (10 × 100 μl PBS) and the radioactivity counted on a gamma counter. The percentage inhibition of membrane IgG or Ab-MTX conjugates was calculated using the following formula:

$$\%\ \text{inhibition} = \frac{\text{c.p.m. (control)} - \text{c.p.m. (anti-membrane IgG or conjugates)}}{\text{c.p.m. (control)}} \times 100$$

The loss of binding activity displayed by the Ab-MTX conjugates was then calculated from the following formula:

$$\%\ \text{loss of inhibition} = \frac{\%\ \text{inhibition (anti-membrane IgG)} - \%\ \text{inhibition (conjugates)}}{\%\ \text{inhibition (anti-membrane IgG)}} \times 100$$

*Assessment of the cytotoxicity of the Ab-MTX conjugates in vitro*

*Estimation of cell number and cell size distribution* The cytotoxicity of the Ab-MTX conjugates was assessed by measuring the number of cells remaining in the cultures after treatment in comparison to controls. Monolayers of PC3 cells were subcultured with trypsin/versene and seeded onto 24-well plates at a density of $5 \times 10^{3}$ cells $ml^{-1}$ DME. The cells were allowed to recover from trypsinisation for 24 h at 37°C before incubation with Ab-MTX conjugates (90 μg Ab $ml^{-1}$, 4 μg MTX $ml^{-1}$), Ab alone (90 μg $ml^{-1}$), MTX alone (0.4-4 μg $ml^{-1}$), Ab plus MTX combined but not conjugated (90 μg Ab $ml^{-1}$, 4 μg MTX $ml^{-1}$), NIgG-MTX conjugates (83 μg IgG $ml^{-1}$, 4 μg MTX $ml^{-1}$) in DME, or DME alone (control) for 2 h at 37°C, using quadruplicate cultures per experimental group. After this time medium was aspirated from the wells, the cultures washed with PBS (5 × 2 ml), fresh medium (1 ml) added to each well and the cultures incubated for 72 h at 37°C. Cells were counted at the end of this period by disrupting the monolayers with trypsin/EDTA and diluting the cells with Isoton (15 ml). The suspended cells were counted on a Coulter counter (model ZB1) equipped with a multichannel analyser facility and an x-y plotter. Total cell population per well was determined by taking account of machine coincidence error, sample volume and dilution of the suspension. Any accumulation of cells within a certain phase of the cell cycle was determined by analysis of the cell size distribution of each experimental group using the multichannel analyser.

*Release of $^{51}$Cr from pre-loaded cells* Release of $^{51}$Cr from pre-loaded PC3 cells was measured by a modification of the method described by Wigzell (1965), and gives an estimation of cell death. PC3 cells were seeded onto 24-well plates, at a density of $5 \times 10^{3}$ cells $ml^{-1}$ and allowed to recover from trypsinisation for 24 h at 37°C. Pre-labelling of the cells was carried out by the addition of $^{51}$Cr in DME (20 μCi

(0.74 MBq) × $10^6$ cells) for 30 min and each well washed individually with PBS (6 × 2 ml). Monolayers of PC3 cells were incubated with Ab–MTX conjugates (90 µg Ab $ml^{-1}$, 4 µg MTX $ml^{-1}$), Ab alone (90 µg $ml^{-1}$) MTX alone (0.4–4 µg $ml^{-1}$), Ab plus MTX combined but not conjugated (90 µg Ab $ml^{-1}$, 4 µg MTX $ml^{-1}$), NIgG–MTX conjugate (83 µg IgG $ml^{-1}$; 4 µg MTX $ml^{-1}$) in DME, or DME alone (control) for 2 h at 37°C. After this time the media was aspirated from the cells, each well washed with PBS (5 × 2 ml) and fresh medium added (1 ml) to each culture. The $^{51}Cr$ released from the cells was measured 24 h later by removal of the media, washing each well individually (2 × 2 ml DME) and counting the total radioactivity in the medium from each well on a gamma counter.

*Assessment of the cytotoxicity of Ab–MTX conjugates in vivo*

The anti-tumour activity of the Ab–MTX conjugates was assessed *in vivo* by their action on the growth of PC3 tumours in athymic nude mice. Mice bearing subcutaneous tumours (0.3–1.1 cm diameter) were divided into six groups (16 animals per group) and injected with Ab–MTX conjugates, Ab alone, MTX alone, Ab + MTX combined but not conjugated, NIgG–MTX conjugates or saline. Drug and antibodies were administered i.m. every 48 h for 12 days at a dose of 1 mg $kg^{-1}$ $^3H$-MTX (22 µg per animal) in saline (100 µl) and an antibody concentration equivalent to that found in the Ab–MTX conjugate (approximately 498 µg per animal). Tumour size was recorded every 2 days as the mean of two perpendicular diameters, one across the greatest width, and the tumour volume estimated using the formula $4/3\,\pi r^3$, where $r$ is the mean radius. Tumour volume calculated in this way was found to have a linear relationship with both tumour weight and tumour volume as measured by water displacement. The errors involved in using this formula were found to be negligible.

*Tissue distribution of $^3H$ in animals treated with Ab–MTX conjugates*

Twenty-four hours after the sixth injection of the animals under study, the animals were anaesthetised, bled by cardiac puncture and killed by cervical dislocation. The tumour and various body tissues were excised and known amounts (up to 100 mg wet weight) were individually placed in counting vials. Tissues were solubilised by the addition of Soluene (1 ml) and incubated at room temperature for up to 48 h before the addition of Dimilume scintillation fluid (5 ml). The radioactivity in each sample preparation was determined by scintillation counting and quenching corrected for by internal standardisation. The concentration of MTX in the tissues was estimated per g wet weight of tissue by extrapolation from the specific activity of the starting material of $^3H$-MTX (4.2 µCi (0.155 MBq) $mg^{-1}$).

*Statistical evaluation*

A Mann–Whitney U test was used to compare the treatment data derived from $^{51}Cr$ release and cell population studies *in vitro* and also from the tissue distribution of $^3H$-MTX from the various treatment groups studied *in vivo*. Tumour volume data were analysed by two-way analysis of variance (ANOVA) providing values for an F statistic and hence a $P$ value, reflecting the level of statistical significance.

### Results

*Conjugation procedures and assessment of antibody and drug activity*

Methotrexate was conjugated to anti-membrane IgG and non-immune IgG via an active ester intermediate. The extent of substitution was 14.6 mol of MTX per mol of IgG with a 64% recovery and a 27.5% loss in immunological activity, as assessed by a competitive binding assay (Table I).

The immunoglobulin fraction used in this study was not completely specific for prostatic tissue as assessed by immunocytochemical techniques. At an antibody dilution of 1/500 (PBS, pH 7.5), human breast, bladder, kidney and intestinal epithelium also exhibited some staining although with a much lower intensity than that observed with benign and carcinomatous prostatic tissue. Staining was always associated with the epithelial cell membrane and no cross-reactivity was observed with any mouse or rat tissues. When equivalent concentrations of Ab and Ab–MTX conjugates were compared in the immunocytochemical procedure, no diminution in membrane staining or change in tissue specificity of the antibody was observed after conjugation (data not shown).

Comparison of free MTX and Ab–MTX conjugates to inhibit the enzyme activity of DHFR indicated that the MTX within the conjugate retained approximately 68% of its ability to bind to the enzyme at a drug concentration of $3 \times 10^{-7}$ M (Figure 1).

*Cytotoxicity testing in vitro*

Cytotoxicity testing was carried out using cultured PC3 cells, each experiment being carried out at least three times. Conjugate and drug concentrations were adjusted to 4 µg MTX $ml^{-1}$ unless otherwise stated, and antibody concentrations to 90 µg protein $ml^{-1}$ (equivalent to the amount of Ab in the Ab–MTX conjugates at 4 µg $ml^{-1}$). Incubations with drugs and conjugates were carried out for 2 h at 37°C and the results expressed as the means from quadruplicate cultures.

*Estimation of cell number* The differences in cell population with the various treatments are shown in Figure 2 and are expressed as number of cells per ml of medium. Incubations with increasing concentrations of MTX (0.4–4 µg $ml^{-1}$) produced a progressive decrease in the number of cells, at 4 µg $ml^{-1}$ MTX the value being 14.6% of the control population. The Ab–MTX conjugates reduced the cell population to 14.9% of the control, and no significant difference was observed between the values obtained for Ab–MTX conjugates and free drug (4 µg $ml^{-1}$).

Significantly larger cell numbers were observed ($P < 0.05$) when the PC3 cells incubated with NIgG–MTX conjugates were compared to cells treated with Ab–MTX conjugates. The addition of membrane Ab alone to cultured cells led to a reduction in cell number compared to controls ($P < 0.05$). Cultures incubated with Ab plus MTX, combined but not conjugated, however, displayed a larger cell population than incubation with MTX alone ($P < 0.05$).

*Cell size distribution* As the inhibition of DHFR leads to inhibition of DNA, RNA and protein synthesis (Bertino, 1963), cells treated with MTX would be expected to be

**Table I** Competitive binding of unlabelled anti-membrane antibody or antibody-methotrexate conjugate with $^{125}I$-antibody in a solid-phase assay using PC3 membrane

| Concentration of antibody (µg) | % inhibition of $^{125}I$-antibody binding: MTX–antibody conjugates | % inhibition of $^{125}I$-antibody binding: Antibody | % loss of binding activity in the conjugates |
|---|---|---|---|
| 250 | 48.7 | 66.3 | 26.5 |
| 100 | 40.2 | 56.8 | 29.2 |
| 50 | 39.3 | 52.5 | 25.1 |
| 25 | 31.2 | 45.7 | 31.7 |
| 10 | 28.2 | 37.6 | 25.0 |

PC3 membrane fractions were adsorbed onto microtitre wells (100 µg protein per well) and known amounts (1–250 µg) of Ab–MTX conjugates or Ab were incubated with $^{125}I$-labelled Ab (2 µCi, 7.4 MBq $mg^{-1}$; 100 µg per well) for 3 h at 37°C. After washing, (10 × 100 µl PBS) total radioactivity was assessed per well. Data are expressed as the mean percentage inhibition from 16 experimental wells.

**Figure 1** The inhibition of dihydrofolate reductase (DHFR) by MTX (——) and Ab-MTX conjugates (----). Methotrexate and Ab-MTX conjugates ($2 \times 10^{-8}$ to $3 \times 10^{-7}$ M, 100 μl per tube) were incubated with the enzyme (0.1 units per tube) in the presence of NADPH (16.6 μg) for 15 min at 25°C. Dihydrofolic acid (12.5 μg in 50 μl) was dispensed into each tube and the change in absorbance measured spectrophotometrically at 340 nm after 6 min.

**Figure 2** The effect of the Ab-MTX conjugates on the number of PC3 cells remaining in culture after treatment. PC3 cells ($5 \times 10^3$ per well) were incubated with DME (1 ml) containing MTX (0.4–4 μg ml$^{-1}$), Ab-MTX conjugates (90 μg Ab ml$^{-1}$; 4 μg MTX ml$^{-1}$), NIgG-MTX conjugates (83 μg IgG ml$^{-1}$; 4 μg MTX ml$^{-1}$). Ab alone (90 μg ml$^{-1}$), Ab + MTX (90 μg Ab, 4 μg MTX ml$^{-1}$) or DME alone. Treatments were carried out for 2 h at 37°C, the media removed from each well and replaced with fresh DME (1 ml). After a further incubation of 72 h cells were trypsinised, diluted with Isoton and total cell population per well measured on a Coulter counter. Data represent mean and + s.d. of quadruplicate cultures.

arrested in the S phase of the cell cycle. The distribution of cell size within control and drug treated cultures was expressed as the number of cells against cell volume (Figure 3). Control cultures displayed a distribution pattern as seen

**Figure 3** The distribution of PC3 cell volume *in vitro* within various experimental groups. PC3 cells ($5 \times 10^3$ per well) were incubated with MTX (4 μg ml$^{-1}$), Ab-MTX conjugates (90 μg Ab ml$^{-1}$; 4 μg MTX ml$^{-1}$), NIgG-MTX conjugates (83 μg NIgG ml$^{-1}$; 4 μg MTX ml$^{-1}$) Ab alone (90 μg Ab ml$^{-1}$), or DME alone for 2 h at 37°C. Cultures were washed ($5 \times 2$ ml PBS) fresh media (1 ml) added to each well and the cells incubated for a further 72 h). After trypsinisation cells were diluted with Isoton and cell population size determined on a Coulter counter. Cell volume data was obtained from multichannel analysis of Coulter counts. **a**, Control (——), MTX (.....); **b**, Ab (——), Ab-MTX conjugates (.....), NIgG-MTX conjugates (----).

in Figure 3a and cells treated with Ab alone had an identical profile (Figure 3b). Incubation of the cells with NIgG-MTX conjugates showed a slight shift towards a larger cell volume (Figure 3b), whereas cultures treated with either MTX alone (4 μg ml$^{-1}$, Figure 3a) or Ab-MTX conjugates (Figure 3b) displayed a pronounced shift towards larger cell volumes indicating an accumulation of cells in the S phase of the cell cycle for these latter two treatments.

*The release of $^{51}Cr$ from pre-loaded cells* The release of $^{51}$Cr into the medium from pre-loaded PC3 cells incubated with the various drug concentrations and conjugate are shown in Figure 4 and expressed as d.p.m. ml$^{-1}$. With increasing concentrations of MTX there was a progressive rise in the amount of $^{51}$Cr liberated from the cells, reaching a value 5.6 times higher than the control of 4 μg ml$^{-1}$ of drug. Essentially similar results were observed after incubation of the cells with Ab-MTX conjugates as obtained with free drug. The NIgG-MTX conjugates were not as effective in causing the release of $^{51}$Cr from the cells as the Ab-MTX conjugates ($P < 0.05$), but did display some cell killing activity as the $^{51}$Cr content of the media was significantly larger than that seen in control cultures ($P < 0.01$). Cells treated with membrane Ab plus MTX displayed a significantly lower liberation of $^{51}$Cr than that observed with free MTX alone ($P < 0.05$). Incubation of the cultured cells with membrane Ab alone demonstrated a higher release of $^{51}$Cr into the medium than determined in medium from control cultures ($P < 0.05$).

**Figure 4** The effect of the Ab–MTX conjugates on the liberation of $^{51}$Cr from pre-loaded PC3 cells *in vitro*. PC3 cells (5 × $10^3$ per well) were incubated with $^{51}$Cr (20 μCi (0.74 MBq) × $10^6$ cells) for 30 min at 37°C before treatment with MTX (0.4–4 μg ml$^{-1}$), Ab–MTX conjugates (90 μg Ab, 4 μg MTX ml$^{-1}$), NIgG–MTX conjugates (83 μg IgG, 4 μg MTX ml$^{-1}$), Ab alone (90 μg ml$^{-1}$), Ab + MTX (90 μg Ab, 4 μg MTX ml$^{-1}$) or DME alone. Incubations were carried out for 2 h at 37°C, the cells washed (5 × 2 ml PBS) and fresh media (1 ml) added to each well. The media was removed from each well 24 h later and the radioactivity measured on a gamma counter. Data represents the mean + s.d. from eight wells per experimental group.

### *Assessment of the cytotoxicity of Ab–MTX conjugates in vivo*

Measurement of the size of individual PC3 tumours growing in mice receiving the various drugs and conjugates was carried out over a 12-day period and is expressed as the percentage change in tumour volume against time (Figure 5). Control animals, treated with saline only, showed a 15-fold increase (mean from 16 animals) in tumour volume over the 12-day period studied. Although NIgG–MTX conjugates appeared to suppress tumour growth, analysis of variance on the tumour data showed no significant difference from controls. Injections of MTX alone, Ab alone or Ab plus MTX resulted in a significant inhibition of tumour growth when compared to the control group ($P < 0.002$). Animals treated with Ab–MTX conjugates, however, displayed an inhibition of tumour growth that was significantly greater than all other treatment groups ($P < 0.001$).

**Figure 5** The effect of Ab–MTX conjugates on the growth of PC3 tumours *in vivo*. Athymic nude mice bearing subcutaneous PC3 tumours (0.3–1.1 cm diameter) were injected i.m. with MTX, Ab–MTX conjugates, NIgG conjugates, Ab alone, Ab + MTX or saline every 48 h over a period of 12 days. Each dose was adjusted to 1 mg MTX kg$^{-1}$ body weight (22 μg per animal in 100 μl saline) and the amount of Ab given to the control groups was equivalent to the concentration of Ab present per 22 μg of MTX in the Ab–MTX conjugates. Tumour size was measured with calipers before each injection and tumour volume calculated from the formula $4/3\ \pi r^3$. Data are expressed as a percentage increase in tumour volume from day 0, each value representing the mean from 16 experimental animals, and the standard errors of the mean.

### *Distribution of $^3$H-MTX in vivo*

The Ab–MTX conjugates, NIgG–MTX conjugates and free MTX given to animals all contained $^3$H-MTX (4.2 μCi (0.155 MBq) mg$^{-1}$ MTX). Measurement of the label was undertaken in five animals from each treatment group in order to assess the uptake and distribution of the tritium label within the animals. The results for each tissue and treatment are expressed as ng MTX g$^{-1}$ wet weight of tissue and also as tissue:blood ratios (Table II). The highest accumulation of radioactivity was observed in the tumours of animals treated with Ab–MTX conjugates, the tumour: blood ratio being 59.7, compared with 7.4 for animals treated with MTX alone, 6.6 for those given Ab plus MTX and 6.3 for those receiving NIgG–MTX conjugates. The results show (Table II) that the distribution of label in the lung, heart, kidney, liver, spleen and skin vary slightly, but not significantly between the treatment groups.

Significantly higher levels of radioactivity ($P < 0.05$) were observed within the blood of animals given Ab–MTX conjugates or NIgG–MTX conjugates compared to those receiving free drug indicating a difference in the rate of clearance.

### Discussion

To maintain therapeutic effectiveness of cytotoxic drug linked to antibodies, conjugation should not lead to a substantial loss in either drug activity or in antibody binding and specificity. Therefore, in this present study the resulting conjugation of methotrexate with a polyclonal antiserum raised against a prostatic tumour cell line PC3 was assessed in several systems which examined both the drugs cytotoxic ability *in vitro* and *in vivo* and the localisation *in vivo*.

The retention of antibody activity after conjugation with

**Table II** Tumour and tissue distribution of $^3$H-MTX and conjugated $^3$H-MTX *in vivo*

| | *Tissue distribution of $^3$H-MTX, ng g$^{-1}$ wet weight* | | | |
|---|---|---|---|---|
| *Tissue* | *MTX* | *Ab + MTX* | *MTX–Ab conjugates* | *MTX–NIgG conjugates* |
| Lung | 290.2 ( 6.9) | 244.9 ( 5.0) | 185.9 ( 2.1) | 201.7 ( 2.1) |
| Heart | 394.4 ( 9.4) | 234.1 ( 4.8) | 156.8 ( 1.8) | 234.4 ( 2.4) |
| Kidney | 718.5 (17.1) | 474.6 ( 9.7) | 1064.0 (12.2) | 972.7 (10.1) |
| Liver | 906.5 (21.6) | 777.9 (15.9) | 1063.7 (12.2) | 1674.4 (17.4) |
| Spleen | 185.4 ( 4.4) | 353.8 ( 7.2) | 293.7 ( 3.4) | 471.3 ( 4.9) |
| Skin | 230.8 ( 5.5) | 293.0 ( 6.0) | 306.2 ( 3.5) | 619.4 ( 6.4) |
| Tumour | 312.1 ( 7.4) | 323.8 ( 6.6) | 5214.8 (59.7) | 604.2 ( 6.3) |
| Blood | 41.9 | 48.8 | 87.4 | 96.4 |

Athymic nude mice bearing palpable PC3 tumours (0.3–1.1 cm diameter) were injected with MTX, Ab + MTX conjugates, NIgG–MTX conjugates or Ab + MTX every 48 h over a period of 12 days. Each dose of MTX was adjusted to 1 mg kg$^{-1}$ body weight (22 μg per animal in 100 μl saline) and the concentration of Ab given to control animals adjusted to the equivalent amount in the Ab–MTX conjugate (498 μg per animal in 100 μl saline). Animals were bled by cardiac puncture 2 h after the last injection, killed and the various body tissues removed. Tissues (up to 100 mg wet weight) were solubilised with Soluene (1 ml) and the amount of $^3$H label measured by scintillation counting. Quench correction was carried out by internal standardisation and the total amount of MTX present calculated with reference to the original starting material (4.2 μCi(0.155 MBq) mg$^{-1}$). Data are expressed as ng MTX per g wet weight of tissue each value representing the mean from five experimental animals. Tissue: blood ratios are given in parentheses.

the least interruption in structure and activity is clearly of major importance, excess drug linkage or adverse reaction conditions leading to denaturation or aggregation of the antibody. In the studies described in this paper, conjugation by an active ester intermediate method produced a molar incorporation ratio of drug to antibody of 14.6 with a 72.5% retention of antibody binding, as estimated by quantitative membrane binding analysis and no detectable change in membrane specificity as assessed by immunocytochemical techniques. A similar molar ratio was reported for conjugation of methotrexate with a monoclonal antibody against prostatic acid phosphatase which retained over 90% of its antibody activity (Deguchi *et al.*, 1986). Such differences may be explained by the availability of amino groups on the particular immunoglobulins under study, an idea supported by the results of Kanellos *et al.* (1985), who noted that although conjugation of methotrexate to two separate monoclonal antibodies produced the same molar ratio of drug and antibody their retention of antibody activity was markedly different.

Assessment of drug activity by the inhibition of dihydrofolate reductase showed some loss in the capacity of methotrexate to inhibit the enzyme when conjugated compared to an equimolar amount of free drug (Figure 1). Such losses of dihydrofolate reductase inhibition have been reported previously when methotrexate was conjugated to several different macromolecules (Deguchi *et al.*, 1986; Fung *et al.*, 1979; Garnett *et al.*, 1983; Kanellos *et al.*, 1985; Kulkarni *et al.*, 1985; Tsuro *et al.*, 1980) and is probably due to the stearic interference of the macromolecule with the binding of the pteridine moiety of the methotrexate to the active site of the enzyme (Baker, 1969). Studies suggest that a reduced ability to inhibit dihydrofolate reductase activity also occurs within the cell. Deguchi *et al.* (1986) observed that methotrexate conjugated to immunoglobulin was less effective than free drug at inhibiting the incorporation of $^{3}$H-deoxyuridine by LNCaP cells in culture, even though methotrexate in the conjugated form was taken up to a greater extent than free methotrexate. This finding is supported by Uadia *et al.* (1985), who showed that when human melanoma cells were treated with $^{3}$H-methotrexate–antibody conjugates, 35% of the radioactivity was found in the low molecular weight fraction and the methotrexate derivatives in this fraction were no more potent inhibitors of dihydrofolate reductase than the original conjugate. Their study suggests that the methotrexate derivatives digested within the cell are biologically less potent than free drug.

In our tissue culture experiments, determination of the cell volume distribution patterns (Figure 3) revealed that the antibody methotrexate conjugate displayed the same profile as methotrexate alone. As the inhibition of dihydrofolate reductase by methotrexate ultimately results in the cessation of purine and amino acid synthesis (Bertino, 1963), the altered distribution pattern displayed by the cultures incubated with free drug compared to control cultures would be expected, reflecting the accumulation of arrested cells in the S phase of the cell cycle. The similar profiles obtained with cells treated with both the antibody–methotrexate conjugates and with free drug would therefore indicate that the methotrexate moiety of the conjugate was still able to inhibit dihydrofolate reductase activity within the cell and hence arrest the cells in S phase, even though potency of the drug may be reduced.

Conjugation of cytotoxic drugs to non-immune IgG have been shown to produce a greater inhibition of tumour cell growth than free drug alone (Hurwitz *et al.*, 1979; Johnson *et al.*, 1981; Kulkarni *et al.*, 1985). In the experiments reported in this paper, non-immune IgG–methotrexate conjugates were significantly less effective than either methotrexate alone or antibody–methotrexate conjugates in reducing the cell number (Figure 2) and causing in the release of $^{51}$Cr from cells *in vitro* (Figure 4). Furthermore, these non-immune conjugates did not significantly reduce tumour growth *in vivo* (Figure 5). Analysis of cell size distribution after incubation of the PC3 cells with the various drugs and conjugates demonstrated that the non-immune methotrexate conjugates failed to display the same distribution profile observed with free drug or antibody–methotrexate conjugates (Figure 3). Assuming that both immunoglobulin conjugates are broken down within the cell in the same manner, the results would suggest that the non-immune conjugates are unable either to bind or to enter the cell to the same extent. Such non-immune immunoglobulin conjugates appear to rely upon non-specific uptake by the cell and discrepancies in data from different laboratories may be due to the amount of non-specific binding regions on the cell surface or the pinocytoic activity of the cell line under study. An altered clearance rate may also contribute to differences found *in vivo*.

Specific antibody–methotrexate conjugates have been reported to enter the cell as a consequence of antibody binding and subsequent endocytosis (Smyth *et al.*, 1987) rather than via the active carrier-mediated transport system used for methotrexate or by the non-specific uptake of non-immune conjugates. Assessments of the cytotoxic effectiveness of the antibody–methotrexate conjugates *in vitro*, using measurements of both $^{51}$Cr release from pre-loaded cells (Figure 4) and cell population parameters (Figure 2) demonstrated that these conjugates were not significantly different in action from an equimolar amount of free drug, even though the dihydrofolate reductase inhibitory activity of the conjugates was reduced. It would appear therefore that the membrane antibody–methotrexate conjugates are efficiently incorporated into the cells after interaction of the antibody with the cell surface and that the methotrexate from the conjugates is sufficient to cause inhibtion of dihydrofolate reductase.

When a drug and tumour specific antibodies have been admininstered in combination, but not conjugated, several workers have observed a more potent cytotoxic action than free drug given alone (Kulkarni *et al.*, 1981; Lee & Hwang, 1979; Tai *et al.*, 1979) and in some cases an enhanced effect has been observed over the conjugate (Hurwitz *et al.*, 1978). No such synergistic effect between drug and antibody could be demonstrated in our study and may be explained by the high affinity of the polyclonal antiserum utilised for the cell membrane. As a carrier mediated active transport system has been reported for methotrexate (Goldman *et al.*, 1968) it is possible that enhanced binding of the antibody to the cell membrane in our study may block this carrier system, thus hampering drug transport. High affinity membrane binding might also explain the cytotoxicity of the antibody when given alone, both *in vitro* (Figures 2 and 4) and *in vivo* (Figure 5). Antibody cytotoxicity is well documented (Motta, 1971) and antibodies against tumour associated cell surface antigens have been demonstrated to produce membrane mediated cell changes either directly (Yang & Vas, 1970) or through complement and other intermediaries both *in vitro* and *in vivo* (Ghose *et al.*, 1977).

When anti-membrane antibody–methotrexate conjugates were given to nude mice bearing PC3 tumours, a highly significant reduction in tumour growth was observed when compared to all other treatment groups (Figure 5). The concentration of $^{3}$H-methotrexate in the tumours of animals given these conjugates were significantly higher than controls ($P<0.01$ in all cases, Table II). Animals treated with both specific anti-membrane antibody and non-immune antibody conjugates displayed significantly higher blood levels of $^{3}$H-methotrexate than animals receiving drug in its free form thus indicating a slower clearance rate. Few studies have involved measurement of the *in vivo* distribution of cytotoxic drugs linked to antibodies. Deguchi *et al.* (1986) reported an increase of $^{3}$H in the liver and kidney of animals given a conjugate of methotrexate linked to a monoclonal antibody against prostatic acid phosphatase (PAP). As PAP is a secretory product of prostatic cells it is conceivable that its release into the blood stream is sufficient to elicit the formation of antibody complexes, which would then be taken up by the reticuloendothelial system. This may explain the elevated levels of radioactivity labelled conjugate within the liver and kidneys of their animals in comparison to controls. Selection of an antiserum against prostatic cell membranes for conjugation with a cytotoxic drug, in preference to one against secretory products of the cells, should reduce the possibility of antibody complexes forming within the blood. Indeed in our study treatment with the anti-membrane antibody–methotrexate conjugates significantly increased drug accumulation at the tumour site without increasing the level of drug in the other body tissues, suggesting that uptake by the reticuloendothelial system was not pronounced.

Although it would appear that the anti-membrane antibody–methotrexate conjugates described in this study provided an efficient means for specificity targeting the drug *in vivo*, other anti-membrane antibody conjugates do not appear to produce such significant tumour reduction (Kanellos *et al.*, 1987; Smyth *et al.*, 1986). As the antibody preparation employed in this study consisted of a purified rabbit polyclonal antiserum to prostate membranes, its effectiveness *in vivo* may probably relate to the existence of numerous antibodies to various cell surface antigens. A cocktail of three monoclonal antibodies against the surface of T lymphocytes, linked to the toxin ricin, was found to be more effective in inhibiting T cell proliferation than any single monoclonal antibody (Vallers *et al.*, 1983). The approach of using a battery of antibodies against various antigenic determinants on the cell membrane may therefore provide a more efficient carrier system for drug targeting.

The polyclonal antibody used in this study was, as stated, not specific for its prostate cell target and we are fully aware of the need for a battery of anti-membrane monoclonals preferential to human prostatic epithelial cells for future targeting and clinical studies.

The authors wish to express their gratitude for the generous financial support of the Tenovus Organisation. The expert advice and assistance of Dr M. Morton is also greatly appreciated. Thanks must also be extended to Mr K. Davies and his staff for the excellent care of the animals throughout this study.

### References

BAKER, B.R. (1969). Tissue-specific irreversible inhibitors of dihydrofolate reductase. *Accounts Chem. Res.*, **2**, 129.

BERTINO, J.R. (1963). The mechanisms of action of the folate antagonists in man. *Cancer Res.*, **23**, 1286.

DEGUCHI, T., MING CHU, T., LEONG, S.S., HOROSZEWICZ, J.S. & LEE, C. (1986). Effect of methotrexate–monoclonal anti-prostatic acid phosphatase antibody conjugate on human prostate tumour. *Cancer Res.*, **46**, 3751.

FAHEY, J.L. & TERRY, E.W. (1973). Ion exchange chromatography and gel filtration. In *Handbook of Experimental Immunology*, Weir, D.M. (ed.), p. 8.1. Blackwell Scientific Publications: Oxford.

FALK, L.C., CLARK, D.R., KALMAN, S.M. & LONG, T.F. (1976). Enzymatic assay for methotrexate in serum and cerebrospinal fluid. *Clin. Chem.*, **22**, 785.

FUNG, W.P., PRZYBYISKI, M., RINGSDORF, H. & ZAHARKO, D.S. (1979). In vitro inhibitory effects of polymer-linked methotrexate derivatives in tetrahydrofolate dehydrogenase and murine L5178Y cells. *J. Natl Cancer Inst.*, **62**, 1261.

GARNETT, M.C., EMBLETON, M.J., JACOBS, E. & BALDWIN, R.W. (1983). Preparation and properties of a drug-carrier antibody conjugate showing selective antibody-directed cytotoxicity *in vitro*. *Int. J. Cancer*, **31**, 661.

GHOSE, T., GUELU, A., FAULKNER, J. & TAI, J. (1977). *In vivo* suppression of EL4 lymphoma by rabbit antitumour sera. *J. Natl Cancer Inst.*, **58**, 693.

GHOSE, T. & BLAIR, A.H. (1978). Antibody-linked cytotoxic agents in the treatment of cancer: current status and future prospects. *J. Natl Cancer Inst.*, **61**, 657.

GOLDMAN, I.D., LICHTENSTEIN, N.S. & OLIVENO, V.T. (1968). Carrier-mediated transport of the folic acid analogue, methotrexate, in the L1210 leukaemia cell. *J. Biol. Chem.*, **243**, 5004.

HUNTER, W.M. & GREENWOOD, F.C. (1962). Preparation of iodine 131-labelled human growth hormone of high specific activity. *Nature*, **194**, 495.

HURWITZ, E., MARON, R., BERNSTEIN, A., WILCHECK, M., SELA, M. & ARNON, R. (1978). The effect *in vivo* of chemotherapeutic drug–antibody conjugates in two murine experimental tumour systems. *Int. J. Cancer*, **21**, 747.

HURWITZ, E., SCHECHTER, B., ARNON, R. & SELA, M. (1979). Binding of anti-tumour immunoglobulins and their daunomycin conjugates to the tumour and its metastases. *In vitro* and *in vivo* studies with Lewis lung carcinoma. *Int. J. Cancer*, **24**, 467.

JOHNSON, J.R., FORD, C.H.J., NEWMAN, C.E., WOODHOUSE, C.S., ROWLAND, G.F. & SIMMONDS, R.G. (1981). A vindestine–anti-CEA conjugate cytotoxic for human cancer cells *in vitro*. *Br. J. Cancer*, **44**, 472.

KAIGHN, M.E., SHANKAR NARAYAN, K., OHNUKI, Y., LECHNER, J.F. & JONES, L.W. (1979). Establishment and characterisation of a human prostatic carcinoma cell line (PC-3). *Invest. Urol.*, **17**, 16.

KANELLOS, J., PIETERSZ, G.A. & MCKENZIE, I.F.C. (1985). Studies of methotrexate-monoclonal antibody conjugates for immunotherapy. *J. Natl Cancer Inst.*, **75**, 319.

KANELLOS, J., PETERSZ, G.A., CUNNINGHAM, Z. & MCKENZIE, I.F.C. (1987). Antitumour activity of aminopterin–monoclonal antibody conjugates; *in vitro* and *in vivo* comparison with methotrexate-monoclonal antibody conjugates. *Immunol. Cell Biol.*, **65**, 483.

KULKARNI, P.N., BLAIR, A.H. & GHOSE, T.I. (1981). Covalent binding of methotrexate to immunoglobulins and the effect of antibody-linked drug on tumour growth *in vitro*. *Cancer Res.*, **41**, 2700.

KULKARNI, P.N., BLAIR, A.H., GHOSE, T. & MAMMEN, M. (1985). Conjugation of methotrexate to IgG antibodies and their $F(ab)^2$ fragments and the effects of conjugated methotrexate on tumour growth *in vitro*. *Cancer Immunol. Immunother.*, **19**, 211.

LEE, F.H. & HWANG, K.M. (1979). Antibodies as specific carriers for chemotherapeutic agents. *Cancer Chemother. Pharmacol.*, **3**, 17.

MENOM, M. & WALSH, P.C. (1979). Hormonal therapy for prostatic cancer. In *Prostatic Cancer*, Murphy, G.P. (ed.) p. 175. PSG: Littleton, MA.

MOTTA, R. (1971). Passive immunotherapy of leukaemia and other cancer. *Adv. Cancer Res.*, **14**, 161.

SIBLEY, P.E.C., HARPER, M.E., PEELING, W.B. & GRIFFITHS, K. (1984). Growth hormone and prostatic tumours: localization using a monoclonal human growth hormone antibody. *J. Endocrinol.*, **103**, 311.

SMYTH, M.J., PIETERSZ, G.A., CLASSON, B.J. & MCKENZIE, I.F.C. (1986). Specific targeting of chlorambucil to tumours with the use of monoclonal antibodies. *J. Natl Cancer Inst.*, **76**, 503.

SMYTH, M.J., PIETERZ, G.A. & MCKENZIE, I.F.C. (1987). The mode of action of methotrexate–monoclonal antibody conjugates. *Immunol. Cell Biol.*, **65**, 189.

STERNBERGER, L.A., HARDY, P.H. Jr, CUCULIS, J.J. & MEYER, H.G. (1970). The unlabelled antibody enzyme method of immunohistochemistry. Preparation and properties of soluble antigen–antibody complexes (horseradish peroxidase–anti horseradish peroxidase) and its use in identification of spirochaetes. *J. Histochem. Cytochem.*, **18**, 315.

TAI, J., BLAIR, A.H. & GHOSE, T. (1979). Tumour inhibition by chlorambucil covalently linked to antitumour globulin. *Eur. J. Cancer*, **15**, 1357.

THOM, D., POWELL, A.J., LLOYD, C.W. & REES, A.H. (1977). Rapid isolation of plasma membranes in high yield from cultured fibroblasts. *Biochem. J.*, **168**, 187.

TSURO, T., YAMORI, T., TSUKAGOSHI, S. & SAKURAI, Y. (1980). Enhanced cytocidal action of methotrexate by conjugation to conconavalin A. *Int. J. Cancer*, **26**, 655.

UADIA, P., BLAIR, A.H., GHOSE, T. & FERRONE, S. (1985). Uptake of methotrexate linked to polyclonal and monoclonal antimelanoma antibodies by a human melanoma cell line. *J. Natl Cancer Inst.*, **74**, 29.

VAITUKAITIS, J., ROBBINS, J.B., NIESCHLAG, E. & ROBBINS, G.T. (1971). A method for producing specific antisera with small doses of immunogen. *J. Clin. Endocrinol. Metab.*, **33**, 998.

VALLERS, D.A., ASH, R.C., ZANJAN, E.D. & 5 others (1983). Anti-T-cell reagents for human bone marrow transplantation: ricin linked to three monoclonal antibodies. *Science*, **222**, 512.

WIGZELL, H. (1965). Quantitative filtration of mouse H-2 antibodies using Cr labelled target cells. *Transplantation*, **3**, 423.

YANG, T.J. & VAS, S.M. (1970). Effects of antibodies on L5178Y mouse leukaemia cells cultured *in vitro*. *Cancer Res.*, **30**, 1231.